# Cost-effectiveness of herpes zoster vaccines in the U.S.: A systematic review

**DOI:** 10.1016/j.pmedr.2022.101923

**Published:** 2022-07-22

**Authors:** Neil R. Meredith, Edward P. Armstrong

**Affiliations:** a West Texas A&M University, Department of Accounting, Economics, and Finance, Canyon, TX, USA; b University of Arizona College of Pharmacy, Department of Pharmacy Practice and Science, Tucson, AZ, USA

**Keywords:** Cost-effectiveness, Herpes zoster vaccine, Systematic review, United States

## Abstract

The purpose of this study was to conduct a systematic review to evaluate the cost-effectiveness evidence of herpes zoster vaccines in the U.S. A systematic literature review was undertaken for U.S. studies focused on the cost-effectiveness of herpes zoster vaccines. Eligibility criteria included studies that evaluated the cost-effectiveness of the recombinant zoster vaccine (RZV) and zoster vaccine live (ZVL) and were published between 2015 and 2021. Article titles and abstracts were reviewed to identify relevant publications. The Consolidated Health Economic Evaluation Reporting Standards (CHEERS) criteria for economic evaluations were used to evaluate the studies. Eleven published studies met inclusion and exclusion criteria. Seven studies compared RZV and ZVL. Four studies compared ZVL dosing regimens with or without a no vaccine option. All studies incorporated health system costs. Ten out of eleven (90.9%) studies conducted their analyses from a societal perspective and included indirect costs. For measurements of effectiveness, ten of eleven (90.9%) studies estimated quality-adjusted life years, four (36.4%) used shingles cases averted, two (18.2%) employed deaths prevented, and one (9.1%) measured life years saved. All studies that compared RZV with no vaccine found RZV to be a cost-effective strategy to prevent both shingles and post-herpetic neuralgia. Additionally, these analyses showed that RZV consistently dominated ZVL. Compliance with the second RZV dose was important for full benefit of the vaccine. The studies identified in this systematic review identified well-constructed cost-effectiveness analyses of herpes zoster vaccines in the U.S. RZV was more cost-effective than no vaccine or ZVL. This systematic review supports removal of ZVL from the U.S. market.

## 1 Introduction

Herpes zoster is the virus responsible for causing chicken pox and often leads to shingles later in life (Solomon and Cohen, 2013). Shingles is well-known to produce significant pain and discomfort, which often lasts for months to years and may lead to post-herpetic neuralgia (PHN) (Yawn et al., 2007). Shingles can occur anywhere on the body, including blisters on the sides (Oster et al., 2005). Patients experience pain, burning, sensitivity to touch, fluid-filled blisters that may crust over, and have itching (Solomon and Cohen, 2013). For many patients the pain is substantial (Yawn et al., 2007, Oster et al., 2005). Anyone who has had chicken pox may develop shingles as the virus enters the nervous system and lies dormant for years (Solomon and Cohen, 2013). Eventually, the herpes zoster virus may reactivate and travel along neural pathways to the skin to produce shingles (Yawn et al., 2007). In addition, after a shingles episode has resolved, some patients may develop PHN from their shingles episode. PHN is a disorder impacting the nerves and skin that produces burning pain that persists after the rash and blisters of shingles has resolved. Risk factors for developing shingles include age 50 and older, weakened immune system including cancer, undergoing treatments such as radiation or chemotherapy, or taking medications such as organ transplant rejecting drugs (Solomon and Cohen, 2013, Yawn et al., 2007). To prevent shingles, herpes zoster vaccinations have been developed. Zoster vaccine live (ZVL) and the recombinant zoster vaccine (RZV) have been clinically evaluated (Lal et al., 2015). Since ZVL was withdrawn from the U.S. market in 2020, there is real interest in better understanding the cost-effectiveness of RZV. Therefore, the goal of this study was to conduct a systematic review to evaluate the cost-effectiveness evidence of the RZV herpes zoster vaccine in the U.S.

## 2 Methods

This study was exempt from review by the West Texas A&M University Institutional Review Board because the study was based on publicly available anonymized databases. This analysis followed the guidance by Mandrik et al. that identified best practices for systematic reviews of cost-effectiveness analyses (Mandrik et al., 2021). Database services of Embase, MEDLINE, and PubMed were searched to locate studies evaluating the cost-effectiveness of herpes zoster vaccines. Economic and zoster terms were expanded and used jointly to capture relevant articles. Search limits were applied to provide the list of article titles for further consideration. Important terms and filters (human research, years 2015–2021, and published in English) used for Embase, MEDLINE, and PubMed literature searches are summarized in Table 1. In addition to conducting searches of electronic abstracting databases, the references of relevant primary studies, guideline documents, published meta-analyses, and authoritative clinical reviews were examined to identify other potential cost-effectiveness articles. The Preferred Reporting Items for Systematic Reviews and Meta-Analysis (PRISMA) guidelines were followed in this analysis (Page et al., 2021).

**Table 1:** Literature search terms used.

	Embase and MEDLINE	PubMed
Herpes zoster vaccine cost-effectiveness	('varicella zoster vaccine'/exp OR 'varicella zoster vaccine') AND ('cost effectiveness analysis'/exp OR 'cost effectiveness analysis')	(“Herpes Zoster Vaccine”[Mesh]) AND “Cost-Benefit Analysis”[Mesh]
Search limits	Articles, humans, 2015–2021, English	Humans, 2015–2021, English
Date last searched	December 22, 2021	December 22, 2021

Studies were eligible for inclusion if they were cost-effectiveness analyses of herpes zoster vaccines. The two study authors worked independently to review the identified studies. In no case did the study authors need to contact the original publication authors to confirm study data. The automated tools in Embase and MEDLINE as well as PubMed were used to find the published manuscripts to be screened. The automated tools identified articles, human studies, manuscripts published between 2015 and 2021, and those published in English. Studies of aggregate cost data such as cost of illness analyses were excluded. Also, cost-effectiveness analyses from countries outside the U.S. were excluded because health systems costs vary widely between countries and have limited generalizability. After completing the searches from abstracting services, titles of identified studies were examined for relevance. Studies appearing to meet inclusion criteria were further reviewed by evaluating the study abstract. Full manuscripts of potentially relevant articles were retrieved to verify eligibility and undergo further data extraction. Studies that included only ZVL and no treatment were excluded because ZVL has been removed from the U.S. marketplace. Two reviewers independently conducted the literature searches and independently reviewed article titles and abstracts for possible inclusion in the analysis. Any differences between reviewers were resolved by reviewing studies together and including or excluding a study based on the inclusion and exclusion criteria. Identified cost-effectiveness studies were then critiqued using the Consolidated Health Economic Evaluation Reporting Standards (CHEERS) criteria (Husereau et al., 2013). The proportion of CHEERS criteria met by each cost-effectiveness publication was determined.

## 3 Results

**Fig. 1:**
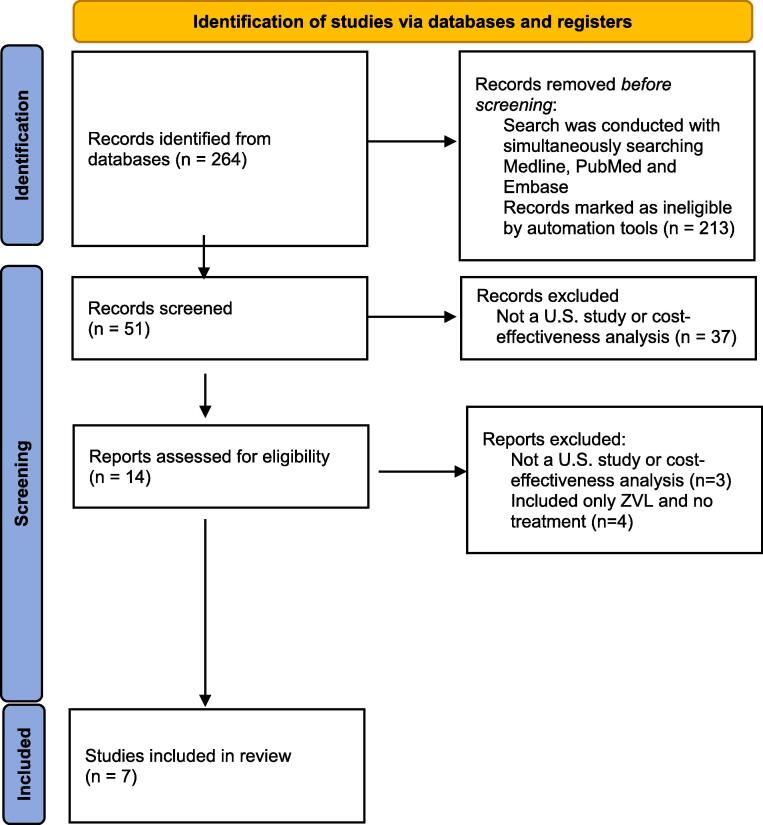
Flow diagram for systematic review processes.

Fig. 1 displays the PRISMA flow diagram describing the article selection process. A total of eleven published studies assessing the cost-effectiveness of herpes zoster vaccines met the inclusion and exclusion criteria (Wilson et al., 2020, Carpenter et al., 2019, Curran et al., 2019, Prosser et al., 2019, Curran et al., 2018, Dooling et al., 2018, Le and Rothberg, 2018, Le and Rothberg, 2018, Le and Rothberg, 2017, Le and Rothberg, Feb 2017, Le and Rothberg, 2015). Four studies exclusively used ZVL, which has been removed from the U.S. marketplace. Nine citations were cost-effectiveness publications. One study was a set of herpes zoster vaccine guidelines that included herpes zoster vaccine original cost-effectiveness research data. One letter to the editor contained herpes zoster vaccine original cost-effectiveness research data.

Table 2 summarizes the characteristics of the identified studies. All studies were conducted from a U.S. perspective. All seven studies with RZV included ZVL as a comparator. All studies incorporated health system costs. Ten out of eleven (90.9%) studies conducted their analyses from a societal perspective and included indirect costs. For measurements of effectiveness, ten of eleven (90.9%) studies used quality-adjusted life years (QALYs), four of eleven (36.4%) studies used shingles cases averted, two of eleven (18.2%) studies employed deaths prevented, and one (9.1%) study used live years saved (LYS). Four of the studies compared different ZVL regimens with or without a no vaccine option and were excluded from further analysis because of removal from the U.S. marketplace.

**Table 2:** Characteristics of the identified cost-effectiveness studies.

Study feature	Number of Studies
Published articles or letters	11 (RZV or ZVL)
Country perspective	11 U.S.
Compared RZV vs. ZVL	7 studies
Compared ZVL regimens with or without no vaccine option	4 studies (excluded from further analysis)
Costs included	10 direct medical + indirect costs (societal perspective) 1 direct medical costs only
Effectiveness endpoint	10 quality-adjusted life years (QALYs) 4 shingles cases averted 2 deaths prevented 1 life years saved (LYS)

RZV = recombinant zoster vaccine, ZVL = zoster vaccine live.

Six of seven (85.7%) studies used a Markov model structure to determine cost-effectiveness. All six used a lifelong time horizon for their Markov models. However, the shingles guideline publication that included original cost-effectiveness research data did not present the model framework used in the analysis (Dooling et al., 2018). In addition, analysis of the identified publications noted that a probabilistic sensitivity analysis was performed in seven of seven (100%) studies. Table 3 summarizes the cost-effectiveness model attributes, funding, and the proportion of CHEERS criteria met by each study. Six of seven (85.7%) studies met at least 90% of the CHEERS criteria, suggesting that most studies were of high quality. Table 4 summarizes the cost-effectiveness analysis findings. Each cost-effectiveness model was unique in evaluating specific comparators, model structure, and outcomes assessed.

**Table 3:** Cost-effectiveness model attributes, funding, and CHEERS criteria.

Author	Year	Full study or letter to the editor	Comparators	Health outcomes	Choice of model	Probabilistic Sensitivity Analysis	Funding Source	CHEERS Criteria Percentage
Carpenter CF, et al.	2019	Full	RZV, ZVL, or no vaccine	costs and QALYs	Markov	Yes	No extramural funding	100.0%

Curran D, et al.	2019	Full	RZV, revaccination with ZVL, or no further vaccination	shingles cases averted, deaths, costs, life years, and QALYs	Markov	Yes	GlaxoSmithKline	96.0%

Prosser LA, et al.	2019	Full	RZV, ZVL, or no vaccine	costs and QALYs	Markov	Yes	Centers for Disease and Control	100.0%

Curran D, et al.	2018	Full	RZV, ZVL, or no vaccine	shingles cases averted, deaths, costs, life years, and QALYs	Markov	Yes	GlaxoSmithKline	96.0%

Dooling KL, et al.	2018	Full	RZV, ZVL, or no vaccine	costs and QALYs	Not stated	Yes	No extramural funding	41.7%

Le P and Rothberg MB	2018	Full	RZV, ZVL, or no vaccine	costs and QALYs	Markov	Yes	No extramural funding	100.0%

Le P and Rothberg MB	2018	Letter	RZV, ZVL, ZVL with RZV, or no vaccine	costs and QALYs	Markov	Yes	No extramural funding	100.0%

QALY = quality-adjusted life year, RZV = recombinant zoster vaccine, ZVL = zoster vaccine live.

**Table 4:** Model findings.

Author	Year Published	Results
Carpenter CF, et al.	2019	In comparison to a no vaccine approach, RZV provided a good value for an array of cost and age at vaccination scenarios. RZV also showed dominance over ZVL in the majority of the various cost and age at scenarios.

Curran D, et al.	2019	Compared to no additional vaccination or revaccination with ZVL, vaccinating U.S. adults 60 + years old, who were vaccinated 5 years prior with zoster vaccine live, with RZV was cost-saving.

Prosser LA, et al.	2019	Compared to no vaccination, RZV ICERs span by age from $10,000 to $47,000 per QALY gained. ICERs were lower than $60,000 per QALY gained for individuals 60 years of age and older. Vaccination with RZV dominated vaccination with ZVL for individuals 60 years of age and older. Cost-effectiveness ratios for RZV were lower than ZVL. The findings were robust for a large variety of possible variable values.

Curran D, et al.	2018	RZV was cost-effective compared to no vaccination for ages 50 and over. The greatest cost savings of vaccinating with RZV occurred at 60 years of age with higher ICERs before and after this age (i.e. 50, 65, 70, and 80 years of age).

Dooling KL, et al.	2018	Comparing no vaccination with RZV, estimates showed that RZV costs $31,000 per QALY gained for immunocompetent individuals 50 years of age or older. To avert one case of PHN and one case of shingles, 70–187 and 11–17 individuals must be vaccinated with RZV, respectively. Receiving RZV 8 weeks after ZVL resulted in values of $15,000 per QALY gained for individuals 80–89 years of age to $117,000 per QALY gained for individuals 50–59 years of age.

Le P and Rothberg MB	2018	RZV was less costly and more effective than ZVL for all immunocompetent individuals aged 60 years or older. In comparison to no vaccination, RZV had an ICER that varied from $20,038 to $30,084 per QALY gained, depending on age at vaccination.

Le P and Rothberg MB	2018	Compared to no vaccination at age 50, RZV had an ICER of $151,430 per QALY gained. A higher adherence rate to the second dose of RZV is more cost-effective at younger ages.

ICER = incremental cost-effectiveness ratio, PHN = post-herpetic neuralgia.

QALY = quality-adjusted life year, RZV = recombinant zoster vaccine, ZVL = zoster vaccine live.

To analyze risk of bias, the identified studies were examined independently by study authors to determine their sponsorship and researchers’ disclosed conflicts of interest. Two of seven studies were funded by pharmaceutical industry. These findings are noted in Table 3. Author conflicts related to study sponsorship were noted in these same two studies. The results reported by studies funded by the pharmaceutical industry were quite similar to the results without this sponsorship. In addition, the direction and magnitude of the cost and effect differences were similar between studies. Across all studies, RZV was consistently a dominant therapy compared to ZVL and a cost-effective therapy compared to no treatment. All 7 studies included probabilistic sensitivity analyses and this strengthens the study conclusions regardless of study sponsorship.

Several studies were especially noteworthy. Carpenter et al. (2019) used a Markov model to compare a 2-dose regimen of RVZ, a 1-dose regimen of ZVL, and a no vaccine strategy . The model included variables for vaccine efficacy, durability of protection, health-related quality of life, resource utilization, costs, and disease epidemiology. The analysis used a U.S. societal perspective and the cycle length was one year with a lifelong time horizon. For individuals vaccinated at age 50 years, RZV produced the greatest reduction in cumulative shingles cases compared to both ZVL and no vaccine (RZV produced a 20.7% reduction compared to no vaccine). RVZ also led to the lowest frequency of PHN cases compared to ZVL and no vaccine (RZV produced an 8.1% reduction compared to no vaccine). RZV also produced slightly more QALYs than ZVL and no vaccine (RZV produced 0.001220 QALYs gained compared to no vaccine). However, RVZ was the most costly regimen compared to both ZVL and no vaccine (RZV was $111.24 more compared to no vaccine). The incremental cost-effectiveness ratio (ICER) was $91,156 per QALY gained for RZV compared to no vaccine. In contrast, if RZV was administered to patients at 60 years of age, the ICER decreased to $19,300 per QALY gained for RVZ compared to no vaccine. If RVZ was administered to patients at 70 years of age, the ICER was lowered to $1,407 per QALY gained compared to no vaccine. The probabilistic sensitivity analysis demonstrated that RVZ was cost effective in 82% of scenarios at a willingness-to-pay value of $150,000 per QALY gained when RZV was administered to a weighted average of patient ages from 50 to 70 years of age.

Prosser et al. (2019) used a Markov model to compare a 2-dose regimen of RVZ, 1-dose regimen of ZVL, and no vaccine strategies. Vaccination with RZV prevented more episodes of shingles than vaccination with ZVL for all age groups. Compared with no vaccine, RVZ prevented 30% of shingles cases for persons aged 50–59 years and 72% of shingles cases for those aged 80–89 years over the lifetime horizon. Vaccination with RZV yielded lower total costs than vaccination with ZVL for all ages because of higher averted shingles disease costs. Vaccination with RZV or ZVL resulted in higher total costs than no vaccination. From a societal perspective, the ICER for vaccination with RZV compared to no vaccination ranged from $10,000 to $47,000 per QALY gained, depending on age at vaccination. RZV dominated ZVL by being more effective and less costly across all age groups. The probabilistic sensitivity analysis demonstrated that RZV was the preferred treatment strategy in 84% of simulations for persons aged 50 to 59 years and 95% of simulations for those aged 60 to 69 years and more than 99% of simulations for those aged 70 to 99 years age at a willingness-to-pay threshold of $100,000 per QALY gained.

Curran et al. (2018) used a Markov model to compare a 2-dose regimen of RVZ, 1-dose regimen of ZVL, and no vaccine strategies . The compliance with a second dose of RZV was assumed to be 69%. In the base case analysis of 1 million U.S. adults at least 60 years of age, RVZ vaccination would prevent 103,603 shingles cases, 11,197 PHN cases, and 14,455 other complications at an ICER of $11,863 per QALY gained compared to no vaccine. In addition, compared to no vaccine, approximately 99.5% of Monte Carlo simulations found RZV to be cost effective at a willingness-to-pay value of $100,000 per QALY gained. Overall, this analysis demonstrated that vaccinating with RZV was cost-effective compared to no vaccine for ages 50, 60, 65, 70, and 80 years of age.

Le and Rothberg (2018) used a Markov model to compare a 2-dose regimen of RVZ, 1-dose regimen of ZVL, and no vaccine strategies. Their results for patients 60 years of age found that RZV had an incremental cost increase of $93 compared to no vaccine and an incremental increase in QALYs of 0.0031 resulting in $30,084 per QALY gained. RZV dominated ZVL by being more effective and less costly. For patients 70 years of age, the RZV ICER decreased to $20,038 per QALY gained compared to no vaccine. For patients 80 years of age, the RVZ ICER was $21,726 per QALY gained compared to no vaccine. The probabilistic sensitivity analysis found that RZV was cost-effective at a willingness-to-pay value of $100,000 per QALY gained in 78% to 93% of scenarios depending on the age at vaccination. Le and Rothberg (2018) further expanded their analysis by lowering the vaccination age to 50 years of age in a subsequent publication . Compared to no vaccination, RZV had an ICER of $151,430 per QALY gained when administered to patients 50 years of age. A higher adherence rate to the second dose of RZV made RZV more cost-effective at younger ages.

## 4 Discussion

This systematic review identified important cost-effectiveness analyses for the prevention of shingles and PHN episodes. Because these episodes represent significant impairment of health-related quality of life for patients and economic burden to healthcare systems, identification of cost-effective prevention strategies is important (Oster et al., 2005). Although each analysis was unique in terms of design, patient population, and comparators, these analyses found that RZV was more cost-effective than no vaccine across a range of age groups (Carpenter et al., 2019, Prosser et al., 2019, Curran et al., 2018, Le and Rothberg, 2018, Le and Rothberg, 2018). The Dooling et al. (2018) manuscript did not provide methodological details . All studies that compared RZV with no vaccine found RZV vaccination is a cost-effective strategy to prevent shingles and PHN episodes (Carpenter et al., 2019, Prosser et al., 2019, Curran et al., 2018, Le and Rothberg, 2018). Additionally, these analyses demonstrated that RZV dominated ZVL and supported the removal of ZVL from the U.S. marketplace in November 2020 (Prosser et al., 2019). Compliance with the second dose of RZV is important to obtain the full benefits of the vaccine (Le and Rothberg, 2018).

Across studies, some variation was noted in the cost-effectiveness between age categories. Prosser et al. (2019) found it more cost-effective to treat patients with RZV 70 to 79 years of age compared to patients 50 to 59 years of age, compared to no vaccine . Curran et al. (2018) reported that vaccinating at age 60 would lead to cost savings, compared to no vaccine, while vaccinating at age 50 would yield an ICER of $14,916 per QALY gained . Because RZV has an ICER well below a threshold of $100,000 to $150,000 per QALY gained, its use should be encouraged by health systems, clinicians, and advocacy organizations for older Americans. In addition, although the identified studies did not conduct their analyses from a patient perspective, because of its cost-effectiveness in preventing painful shingles or PHN episodes, patients with a high deductible for their prescription drugs may consider RZV vaccination as a reasonable purchase to protect from the adverse clinical sequela of shingles and PHN.

Across all studies, it was impressive that only one study met less than 90% of CHEERS criteria. Additionally, it was encouraging that probabilistic sensitivity analyses were presented in seven of seven (100%) publications. These attributes demonstrate that the identified cost-effectiveness analyses were of high quality and it gives readers confidence in their results supporting the cost-effectiveness of RZV.

There are several important limitations to this systematic review. Although care was taken to identify all published cost-effectiveness analyses of herpes zoster vaccines, it is possible that other studies have been conducted but were not identified and included in this analysis. In addition, this analysis assumes the models were constructed accurately and model variables were populated appropriately by the cited researchers. Despite these limitations, this systematic review clearly demonstrates that RZV is cost-effective compared to no vaccination and the previous product, ZVL.

## 5 Conclusion

The studies identified in this systematic review identified well-constructed cost-effectiveness analyses of herpes zoster vaccines in the U.S. RZV is more cost-effective than no vaccine and the previous product, ZVL. Compliance with the second dose of RZV is crucial. Since RZV is quite cost-effective compared to no vaccine, its use should be encouraged by health systems, clinicians, and advocacy organizations for older Americans.

## Disclosure of ethical compliance

As this is a review of existing original articles, no ethical review was required. The authors have no relevant sources of financial support or potential conflicts of interest to report.

## Funding sources

This research did not receive any specific grant from funding agencies in the public, commercial, or not-for-profit sectors. This research also did not receive any extramural funding.

### CRediT authorship contribution statement

**Neil R. Meredith:** Conceptualization, Methodology, Validation, Formal analysis, Investigation, Resources, Writing – original draft, Writing – review & editing, Visualization. **Edward P. Armstrong:** Conceptualization, Methodology, Validation, Formal analysis, Investigation, Resources, Writing – original draft, Writing – review & editing, Visualization.

## Declaration of Competing Interest

The authors declare that they have no known competing financial interests or personal relationships that could have appeared to influence the work reported in this paper.

## Data Availability

No data was used for the research described in the article.
